# Corrigendum to: Burn Center Organization and Cellular Therapy Integration: Managing Risks and Costs

**DOI:** 10.1093/jbcr/irab130

**Published:** 2021-08-02

**Authors:** Michèle Chemali, Alexis Laurent, Corinne Scaletta, Laurent Waselle, Jeanne-Pascale Simon, Murielle Michetti, Jean-François Brunet, Marjorie Flahaut, Nathalie Hirt-Burri, Wassim Raffoul, Lee Ann Applegate, Anthony S de Buys Roessingh, Philippe Abdel-Sayed

**Affiliations:** 1 Department of Musculoskeletal Medicine, Plastic, Reconstructive and Hand Surgery Service, Lausanne University Hospital, University of Lausanne, Switzerland; 2 Department of Interdisciplinary Centers, Lausanne Burn Center, Lausanne University Hospital, University of Lausanne, Switzerland; 3 Department of Interdisciplinary Centers, Cell Production Center, Service of Pharmacy, Lausanne University Hospital, University of Lausanne, Switzerland; 4 Directorate Department, Unit of Legal Affairs, Lausanne University Hospital, University of Lausanne, Switzerland; 5 Oxford Suzhou Center for Advanced Research, Science and Technology Co. Ltd., Oxford University, Suzhou, PR China; 6 Center for Applied Biotechnology and Molecular Medicine, University of Zurich, Switzerland; 7 Women-Mother-Child Department, Children and Adolescent Surgery Service, Lausanne University Hospital, University of Lausanne, Switzerland

In the original publication of this article, a funding acknowledgement was lacking. The following should have been included: “Acknowledgements: Philippe Abdel-Sayed and Lee Ann Applegate acknowledge funding from the Marie Sklodowska-Curie Action (N°833594—PHAS).” This error is now corrected within the article.

